# Supplementary material for: Chromothripsis during telomere crisis is independent of NHEJ, and consistent with a replicative origin
Source: Genome Res. 2019 May;29(5):737–49. doi: 10.1101/gr.240705.118 (PMC6499312; doi:10.1101/gr.240705.118)
Supplement: Supplemental Material [file supp_gr.240705.118_Supplemental_file_1.zip › contigs/annotated_contigs/DB116/contig.2.DB116_length_377_mean_cov_4.84615384615.docx]

**DB116_length_377_mean_cov_4.84615384615**

```
GGAGGAAGGGAGGAAGGGAGGAGGGAGGAAAGGAGGAGGGAAGGAGGGAGGAAGGGAG|GAGAGAAGGAGGGAGAG|AGGGAGGGAGGA
 >chr20:4008980-4009037 - E=1e-06 p=0e+00                                   >chr8:21390
AGGAAAGGAAGGAAGGAAGGAGTGAGGACTACTGCATTTGTTGCTGCCTCTGAAGGAGCCCAGACAGAGCTGATGGCCCCTGCAGCAAG
33-2139334 - E=9e-160
CGAGACCTACTGTTCACCTGGCAAGGTCCTGTTGTTTGACCAATATTGTCTCCTTCAGTCCTCACAAGTTACAATATATCAGCAAATTG

GAATGGTTTTCATAAGAATAAGTAAAGGAAACATTAGTCATTTATTAAAGAACACAGCAGACACGTGGCCTACTATTATGAGTTATTTG

CATTGTGAAGAAGGATATGAGTA
```
